# Pilot testing of the spring operated wearable enhancer for arm rehabilitation (SpringWear)

**DOI:** 10.1186/s12984-018-0352-4

**Published:** 2018-03-02

**Authors:** Ji Chen, Peter S. Lum

**Affiliations:** 1 Department of Biomedical Engineering, Catholic University of America, Washington, DC 20064 USA; 2 Center for Applied Biomechanics and Rehabilitation Research MedStar National Rehabilitation Hospital, Washington, DC 20010 USA; 3 The Functional & Applied Biomechanics Section Rehabilitation Medicine Department Clinical Center, National Institutes of Health, Bethesda, MD 20892 USA

**Keywords:** Upper extremity, Stroke, Therapy, Exoskeleton, Neurorehabilitation

## Abstract

**Background:**

Robotic devices for neurorehabilitation of movement impairments in persons with stroke have been studied extensively. However, the vast majority of these devices only allow practice of stereotyped components of simulated functional tasks in the clinic. Previously we developed SpringWear, a wearable, spring operated, upper extremity exoskeleton capable of assisting movements during real-life functional activities, potentially in the home. SpringWear assists shoulder flexion, elbow extension and forearm supination/pronation. The assistance profiles were designed to approximate the torque required to move the joint passively through its range. These three assisted DOF are combined with two passive shoulder DOF, allowing complex multi-joint movement patterns.

**Methods:**

We performed a cross-sectional study to assess changes in movement patterns when assisted by SpringWear. Thirteen persons with chronic stroke performed range of motion (ROM) and functional tasks, including pick and place tasks with various objects. Sensors on the device measured rotation at all 5 DOF and a kinematic model calculated position of the wrist relative to the shoulder. Within subject t-tests were used to determine changes with assistance from SpringWear.

**Results:**

Maximum shoulder flexion, elbow extension and forearm pronation/supination angles increased significantly during both ROM and functional tasks (*p* < 0.002). Elbow flexion/extension ROM also increased significantly (*p* < 0.001). When the subjects volitionally held up the arm against gravity, extension at the index finger proximal interphalangeal joint increased significantly (*p* = 0.033) when assisted by SpringWear. The forward reach workspace increased 19% (*p* = 0.002). Nine subjects could not complete the functional tasks unassisted and only one showed improvement on task completion with SpringWear.

**Conclusions:**

SpringWear increased the usable workspace during reaching movements, but there was no consistent improvement in the ability to complete functional tasks. Assistance levels at the shoulder were increased only until the shoulder could be voluntarily held at 90 degrees of flexion. A higher level of assistance may have yielded better results. Also combining SpringWear with HandSOME, an exoskeleton for assisting hand opening, may yield the most dramatic improvements in functional task performance. These low-cost devices can potentially reduce effort and improve performance during task practice, increasing adherence to home training programs for rehabilitation.

## Background

There are 800,000 new strokes in the United States each year [1]. Many survivors experience debilitating motor impairments in the upper extremity that negatively affect functional capacity and quality of life. Impairments can include weakness [2] and lack of coordination between different muscle groups [3]. Fifty percent of stroke survivors older than 64 have persistent hemiparesis at six months post-stroke and 26% are dependent in activities of daily living (ADL) [1]. Unfortunately, a very high level of upper extremity motor control may be needed before the impaired limb is actually incorporated into ADL. Stroke patients often appear to have adequate movement ability when observed in the laboratory, but don’t use the limb with the expected regularity [4].

Neurorehabilitation of these impairments is possible with task-specific repetitive movement practice that incorporates high repetition, volitional effort, and successful completion of tasks to prevent frustration and maintain motivation [5]. Robotics has been studied extensively as a means of assisting movements with forces applied to the limb, allowing completion of movements that would otherwise be impossible to complete unassisted. A recent meta-analysis of 34 studies including 1160 subjects found that robotic devices produced larger gains in arm function, strength and ADL ability than comparison interventions [6]. However, authors concluded the advantages of robotic therapy may not be clinically relevant. The vast majority of these studies involved patients traveling to the clinic on a regimented schedule to practice components of tasks. Home-based approaches may be more effective in increasing the amount of limb use, in particular devices that can assist movements while subjects perform real-world ADL.

Many robotic treatments involve providing partial support of the arm against gravity to enable practice of reaching within a larger workspace [7, 8]. This approach is motivated by research that has shown that many stroke patients have an abnormal synergy whereby elevation of the shoulder against gravity impairs the ability to extend the elbow [9–11]. More recently, work has shown an abnormal coupling of proximal and distal arm muscles, such that activation level of proximal muscles and level of arm support can affect control of hand and wrist muscles [12, 13]. However current robotic approaches that provide gravity compensation do not allow practice of tasks in real-world environments, such as in the home, while standing or when performing bimanual tasks. Also, the provision of gravity support may not completely overcome distal weakness in elbow extension and forearm supination [14]. Additionally, many robotic paradigms rely on repetitive performance of components of functional tasks, for example, planar reaching movements. This contradicts motor learning studies that suggest retention and generalization of skills requires task variability [15–17].

In previous work we developed a wearable passively actuated hand exoskeleton (HandSOME) that increases finger ROM and function [18, 19]. However, some subjects were found to be inappropriate for HandSOME because of proximal weakness, and while the HandSOME enabled adequate range of motion at the fingers, some subjects had difficulty supinating the forearm enough to properly grasp certain objects. Furthermore, finger extension ability would degrade as the arm was lifted against gravity. This motivated the development of a wearable arm exoskeleton called the spring-operated wearable enhancer (SpringWear). Springs apply an angle-dependent torque to the joints, with the goal of increasing ROM, repertoire of possible movements, task variability and success in completing tasks.

Overall, the goal was to enable effective use of the impaired limb in the home environment, thereby allowing patients a highly variable, but meaningful task practice. SpringWear can also reduce effort in task completion promoting greater adherence to home practice schedules. At the shoulder, SpringWear provides partial gravity compensation, which reduces the muscle forces at the shoulder needed to lift the arm. With proper selection of assistance level, the initiation and control of movements are still under patient control but less effort is needed and a larger ROM can be achieved. At the elbow, patients often can flex the joint, but have limited extension, so extension torques are applied to increase elbow extension range. A similar strategy is used to assist forearm supination/pronation. In order to benefit from SpringWear, patients should have some active movement at the joints, but for profoundly weak patients, this approach will not be effective. In these patients, powered exoskeletons may be needed, since they can move the limb throughout a larger workspace than spring powered devices. However, powered devices are much more expensive and complicated to integrate into a wearable device and compact designs are often high impedance, requiring sensors to infer the patient’s intended movement trajectory, which may be difficult in very low level patients.

In this study, chronic stroke patients performed a number of tasks with and without assistance from the SpringWear, and the kinematics of the movements were compared. If successful, SpringWear combined with HandSOME, may provide an inexpensive home-based intervention for a wide range of severe to moderately impaired stroke patients.

## Methods

### SpringWear design

**Fig. 1:**
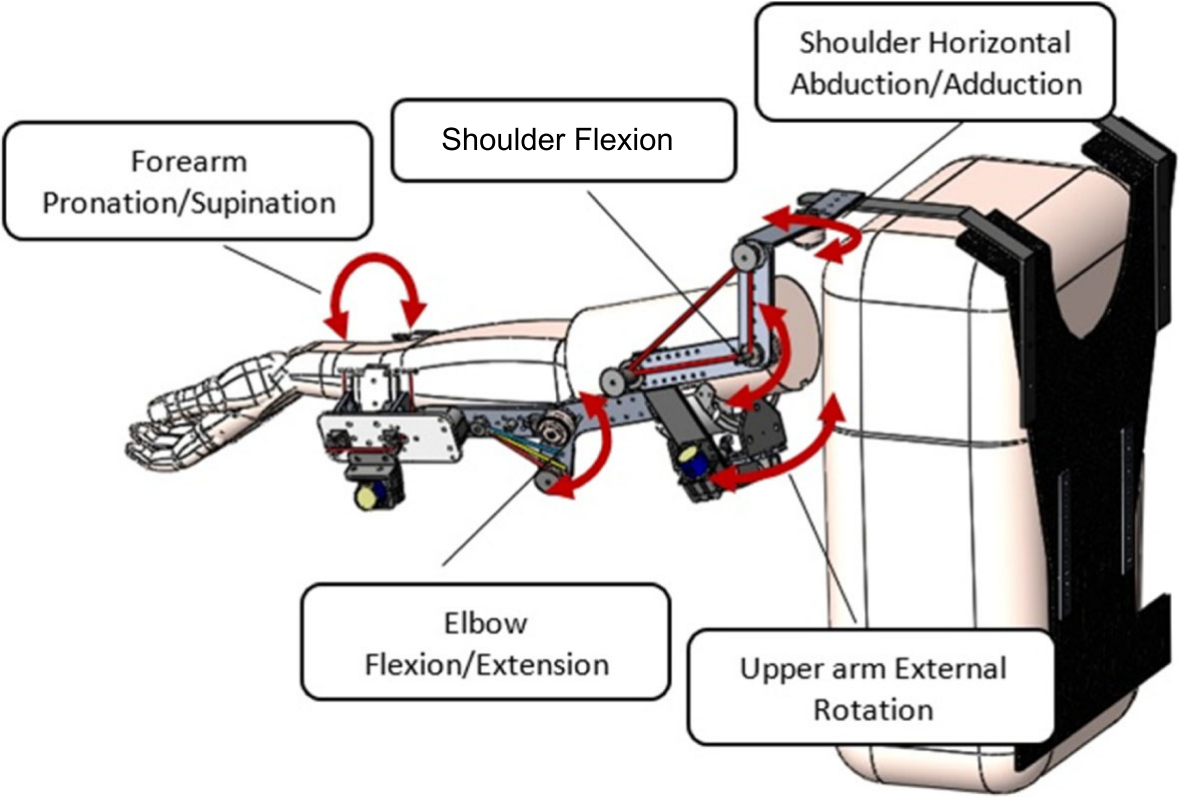
Full Assembly of SpringWear with back splint. Double-headed arrows represents five degrees of freedom. Assistance was applied at shoulder FE, elbow FE, and supination/pronation

An upper limb exoskeleton, in general, needs to be adaptable to different segment lengths and have a high number of DOFs in order to allow realistic movement practice with many anatomical joint axes involved [20]. With five DOFs, SpringWear was designed to provide assistance to forearm supination/pronation, elbow extension, shoulder flexion, while providing passive joints for shoulder horizontal abduction/adduction and internal/external rotation to allow realistic upper limb movements (Fig. 1). The design uses rubber bands or bungee cords as springs to provide the assistance. These profiles can be customized for each subject by adjusting the stiffness of the springs, which is dependent on impairment level.

**Fig. 2:**
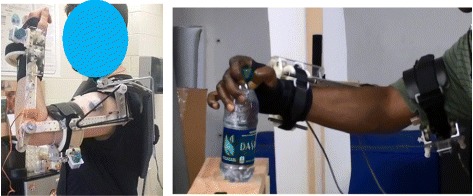
SpringWear worn by subjects. Left: a subject testing SpringWear assistance on his left arm. Right: Stroke subject wearing SpringWear at his right side during functional task performance

During the study, these cords were added together in parallel to obtain the assistance level needed for each joint (a complete description of the procedure is below under Test Protocol). Table 1 gives a summary of exoskeleton torques available from different cord combinations and the maximum and typical human torques used during daily activities [21]. Torque assistance levels available from SpringWear exceed levels used during typical human arm activities. SpringWear weighs 1.2 kg and has the following range of motion (ROM): pronation/supination (PS, 180 degrees), elbow flexion/extension (FE, 120 degrees), shoulder flexion/extension (FE, 120 degrees), upper arm internal/external rotation (90 degrees) and shoulder horizontal abduction/adduction (180 degrees). Hard stops are used to limit the ROM at each joint. A light-weight carbon fiber back splint is worn to support the SpringWear. The arm is connected to the exoskeleton at two points: on the forearm proximal to wrist and on the upper arm proximal to elbow (Fig. 2). Velcro straps and padding were used to comfortably secure the subject’s arm at these points.

**Table 1:** SpringWear joint torque output

Joint	SpringWear Min/Max Torque (Nm)	Typical Human Torque in Daily Life (Nm) [21]	Maximum Human Arm Torque (Nm) [21]
Shoulder FE	4.91/16.7	9.6	115
Elbow FE	0.2/4.3	3.8	72
Forearm PS	0.1/0.5	0.4	9

**Fig. 3:**
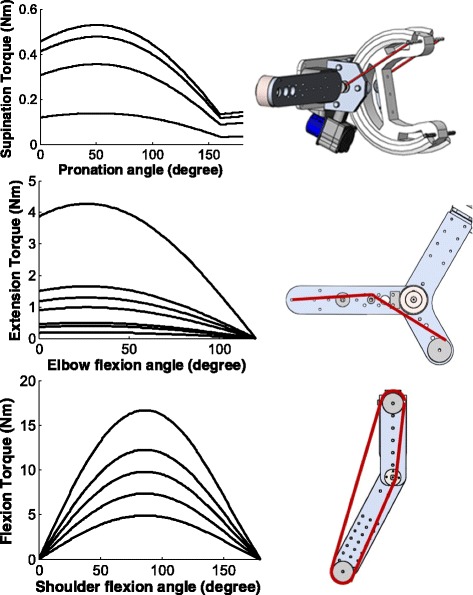
Range of assistance profiles used during testing in supination/pronation, elbow extension, and shoulder flexion. Pictures in the right column show the mechanical structure of these 3 DOF and the spring paths used (red lines)

Details of the design have been presented previously [22]. Briefly, the main mechanical parts of the forearm PS DOF design include a plastic C-shaped collar with 4 V-shaped rails and a compartment with 6 wheels (Bishop-Wisecarver, USA) that guide the rails in a circular arc (Fig. 3). The subject’s forearm proximal to wrist is secured to the opening on the collar. A single elastic cord is routed through pulleys to both sides of the plastic C-shaped collar to assure application of balanced forces. The assistance strategy was similar to what we found effective in the HandSOME device [18], an assistance profile that approximately matches the torque needed to passively move the joint through its range of motion. For most patients, this is a supination assistance torque that is minimal in the fully pronated position, and increases to a maximum at full supination. Fig. 3 shows typical profiles used in testing. The torque profile drops as the joint is moved in pronation, but then increases between 160 and 180 degrees as full pronation is reached. This change in slope is due to the spring wrapping around the forearm splint in this angular range. However, the overall goal of increasing torque as the arm supinates was approximated. The upper arm internal/external rotation mechanism was based on the same design and can provide similar external rotation assistance if needed.

At elbow FE, stroke subjects can have a strong flexion synergy which flexes the elbow and prevents normal extension ROM [9, 10]. We used an assistance strategy similar to that used in forearm PS, with the desired assistance torque maximal at the extended position and minimal at the flexion limit. The mechanical structure is a simple revolute joint constructed from ball bearings and a steel shaft. As the Velcro straps and padding take some space, the flexion limit was set at 120 degree flexion angle. A spring path was selected that produced a roughly monotonic decreasing profile (Fig. 3). A strictly monotonic profile was not possible because of space constraints.

The shoulder FE mechanism was designed to balance the arm against gravity. The design goal was shoulder elevation up to 120-degree ROM. One unique aspect of the design is the wrapping of the elastic cord around 3 pulleys into the shape of a closed triangle (Fig. 3). This approximates a zero rest length spring without requiring overstretching of the elastic cord. For safety, each of the elastic bands were designed to only double in length over the range of motion. Note that the profiles peak at 90 degrees and drop to 0 torque at 0 and 180 degrees shoulder flexion, as is required to match the gravity loading on the arm (Fig. 3).

### Test protocol

This study was conducted at the MedStar National Rehabilitation Hospital. All participants provided informed consent and the protocol was approved by the local human subjects IRB. Thirteen subjects were enrolled into the study. All subjects had a diagnosis of stroke more than six months prior to entry into the study, impaired ability to open the affected hand and difficulty performing reach and grasp tasks. The goal was to evaluate safety, comfort and function of the SpringWear device in chronic stroke patients. Tasks included 4 ROM tests and 3 functional tasks. These tasks provided a systematic evaluation of SpringWear’s effect on movement patterns in chronic stroke subjects.

Four tasks were designed to assess joint ROM with and without spring assistance. Task 1: For shoulder FE, the subjects flexed the shoulder in the frontal plane as high as possible. Task Task 2: For elbow FE, the subjects first stabilized the shoulder at 90 deg. of flexion (or as close to 90 deg. as possible), with the upper arm internally rotated, and then flexed the elbow as far as possible, and then extended the elbow as far as possible. Task 3: For forearm PS, subjects stabilized the shoulder in a neutral position with the elbow flexed at 90 deg., then pronated the forearm as far as possible, then supinated as far as possible. Task 4: For hand ROM, subjects stabilized the shoulder at 90 deg. flexion, then closed the hand and opened the hand as far as possible. The ability of SpringWear to improve performance of functional tasks was examined in three tasks simulating ADL. Task 5: Pick up a water bottle and place it on the shelf. Task 6: Pick up a large horizontally oriented dowel with an overhand pronated grip and place in hole. Task 7: Pick up the dowel with underhand supinated grip and place in hole.

Two testing conditions are used through this study. In the “unassisted” condition, no assistance to movement was provided, but a set of light elastic cords were attached at the shoulder flexion DOF to remove the effect of gravity on the device only. In the “assisted” condition, springs were added according to the following protocol. Springs were added until the subject could voluntarily hold the shoulder at 90 degrees of flexion. Then springs were added to the elbow DOF until the subject could extend the elbow to near full extension while holding the shoulder at 90 degrees of flexion and internally rotated. Finally, springs were added to the forearm SP DOF with the arm held voluntarily in this position, until full supination was possible. For the final 2 subjects, we added one additional condition whereby the springs at the shoulder DOF were added until approximately 100% gravity compensation was achieved and the patient could hold the arm at 90 degree shoulder flexion with little or no effort. This final condition was tested to examine whether or not 100% gravity compensation would significantly increase the finger joint angles. The patient’s body weight was obtained before the test. His or her arm weight was estimated as 5% of body weight [23].

### Data acquisition and analysis

An Arduino-based data acquisition system measured joint rotations at 5 DOFs using small encoders (Contelec GL60, Switzerland) and string potentiometers (Celesco SM1–12, USA). Data was collected from three encoders and two string potentiometers at 30 Hz. Calibration was done on each sensor before each subject testing session. Data was downloaded to a PC for analysis. For Task 4, hand images from video were processed in ImageJ to measure index finger joint angles. Care was taken to keep the plane of index finger movement perpendicular to the camera. The frame with the maximum index finger extension was selected by visual inspection. The index finger angles were measured by drawing lines to represent the finger segments allowing measurement of the metacarpophalangeal (MCP) joint, the proximal interphalangeal joint (PIP) joint and the distal interphalangeal joint (DIP) of the index finger.

Two test trials were performed for each task condition. For the ROM tasks, the main metrics were max shoulder flexion in Task 1, max elbow extension in Task 2, max PS in Task 3, max index finger extension in Task 4. ROM was also calculated as the maximum minus the minimum joint angle. Metrics were averaged across the repetitions at each condition, before comparison between test conditions (with and without spring assistance). For the 3 functional tasks, maximum angles and ROM were calculated for each trial, then averaged across all functional task trials before statistical analysis. The joint angles were also entered into a kinematic model to calculate position of the wrist relative to the shoulder, and the maximum forward reach workspace was also calculated. The Shapiro Wilk test was performed on all data to test normality assumptions before statistical analysis. Two–tailed paired t-tests were used to determine significant differences.

## Results

All thirteen subjects completed the study. During testing of the first three patients, the complaints were difficulty closing the thumb because of the wrist splint on the device and the back splint tended to shift from side to side. These two issues were addressed for the remaining 10 patients. The thermoplastic wrist splint was replaced by a commercial fitted wrist brace attached to the forearm PS mechanism using Velcro. Lightweight polypropylene webbing and plastic buckles were added to make a new harness system that reduced lateral shifting of the back splint. Based on the ROM without assistance, 5 subjects were given assistance in pronation and the rest of the subjects were assisted in supination. The peak of the assistance profiles used for each subject is summarized in Table 2.

**Table 2:** Subject Characteristics

					ROM without assistance (deg.)			Peak assistance torques Applied by SpringWear (Nm)		
No	Male/Female	Age (yrs.)	Months Post	FM	Shoulder FE	Elbow FE	Forearm PS	Shoulder FE	Elbow FE	Forearm PS
1	M	38	58	40	79.46	103.3	118.6	9.8	0.19	0.53
2	M	66	64	36	96.7	95.1	86.0	4.91	0.19	0.53
3	M	57	61	42	87.7	85.6	78.2	4.91	0.24	0.53
4 ^a^	F	43	34	36	55.7	82.9	51.8	7.36	0.40	0.36
5	M	40	37	19	30.9	28.2	24.2	9.8	1.66	0.53
6	F	64	35	27	46.6	54.7	36.2	7.36	1.30	0.36
7 ^a^	F	61	7	20	96.8	89.9	111.4	7.36	0.50	0.36
8	F	44	20	13	6.0	52.9	15.8	9.8	1.66	0.53
9	F	66	48	28	13.3	50.8	67.5	7.36	0.99	0.36
10	M	42	26	N/A	42.3	39.4	21.8	9.8	4.28	0.14
11^a^	M	59	11	29	78.2	91.9	29.9	9.8	4.28	0.53
12	F	78	12	30	17.4	24.1	78.0	16.7	4.28	0.48
13^a^	F	44	38	36	74.7	102.8	92.8	12.3	0.19	0.48

Patients no. 4,7,11 and 3 (marked with^a^) needed assistance to pronate instead of supinate

**Fig. 4:**
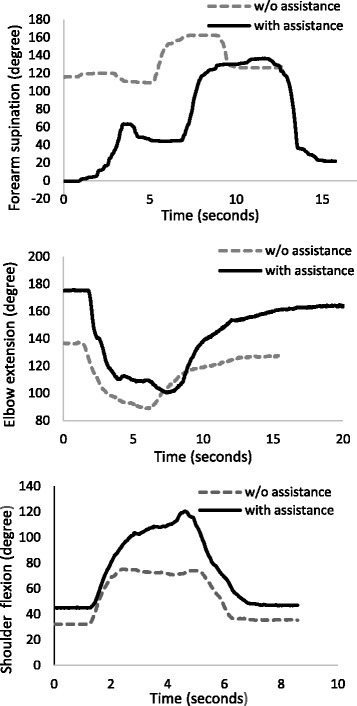
Typical movement data during ROM tasks. The task is to rotate each DOF as far as possible in one direction and then the opposite direction. For this subject, increased ROM was seen at all DOF with assistance. For the elbow, 180 deg is full extension. Full pronation is 0 degree and full supination is 180 degree. This subject had good ability to supinate the forearm and was given assistance in pronation

The testing data from the ROM Tasks 1–4 showed that subjects had immediate gains in assisted DOFs. Typical movement data are presented in Fig. 4 and group averages are summarized in Table 3. With assistance from SpringWear, subjects had significant gains in maximum joint angles at shoulder flexion (gain = 27.6 ± 17.8 degrees; *p* < 0.001), elbow extension (gain = 18.7 ± 13.2 degrees; *p* < 0.001), and forearm PS (gain = 38.8 ± 32.2 degrees; *p* = 0.001). Subjects also had significant ROM gains in elbow FE (gain = 16.9 ± 10.0 degrees; *p* < 0.001), and smaller nonsignificant gains in shoulder FE and forearm PS ROM. Assistance from SpringWear also improved some aspects of index finger control even without directly assisting these DOF. Index finger PIP extension increased significantly with SpringWear assistance (gain = 16.2 ± 20.3 degrees; *p* = 0.033).

**Table 3:** Changes in maximum angle and ROM in ROM tasks

	w/o assistance	Increase with assistance	
	Mean (SD)	Mean (SD)	*p* value
Shoulder flexion max. (deg)	82.6 (25.1)	27.6 (17.8)	**< 0.001**
Elbow extension max. (deg)	142.7 (22.1)	18.7 (13.2)	**< 0.001**
Forearm PS max. (deg)	122.9 (48.6)	38.8 (32.2)	**0.001**
Shoulder FE ROM (deg)	53.7 (31.8)	4.1 (14.2)	0.322
Elbow FE ROM (deg)	69.1 (27.9)	16.9 (10.0)	**< 0.001**
Forearm PS ROM (deg)	61.9 (34.9)	3.0 (31.8)	0.736
MCP extension max. (index finger) (deg)	147.0 (22.5)	−3.1 (13.8)	0.502
PIP extension max. (index finger) (deg)	99.8 (28.5)	16.2 (20.3)	**0.033**
DIP extension max (index finger) (deg)	138.4 (26.5)	7.4 (19.5)	0.261

*SD* is standard deviation. Full extension at elbow, MCP, PIP and DIP joints is 180 degree. Full supination is 180 degree. For patients who needed assistance to pronate instead of supinate, their forearm max pronation angle was measured instead and was converted to have full pronation as 180 degree for statistical analysis. The increase with assistance is averaged across all subjects. The *p* value tests if this increase was significant

Bolded numbers are *p* value less than the significance level 0.05

The gains in the functional tasks were generally consistent with the ROM tasks (Table 4). Subjects had significant gains in maximum shoulder flexion (*p* < 0.001), elbow extension (*p* = 0.002) and forearm pronation/supination (*p* < 0.001) with assistance from SpringWear during functional tasks. The forward reach workspace also increased significantly by 19% (gain = 8.2 ± 7.4 cm; *p* = 0.002). However, the gains in ROM were small and nonsignificant. The success in completing functional tasks was also recorded. Nine subjects could not complete the functional tasks. One subject improved with assistance by completing one trial of Task 6 while not completing any of the trials without assistance. However, there were no other cases of changes from failure to a success when assisted by SpringWear.

**Table 4:** Changes in kinematics in functional tasks

	w/o assistance	Increase with assistance	
	Mean (stdev)	Mean (stdev)	*p* value
Shoulder flexion max. (deg)	69.1 (17.2)	26.0(15.7)	**< 0.001**
Elbow extension max. (deg)	146.4 (19.7)	17.5(14.8)	**0.002**
Forearm PS max. (deg)	107.9 (44.9)	43.5(29.1)	**< 0.001**
Forward reach workspace (cm)	43.8 (5.4)	8.2(7.4)	**0.002**
Shoulder FE ROM (deg)	40.2 (19.5)	3.5(13.6)	0.377
Elbow FE ROM (deg)	46.4 (16.8)	0.7(12.9)	0.858
Forearm PS ROM (deg)	43.8 (25.3)	3.9(24.2)	0.572

Bolded numbers are *p* value less than the significance level 0.05

Subjects 12 and 13 were also tested with 100% arm gravity support, where the shoulder assistance was increased until the subject could relax and the shoulder was fully supported at 90 degrees of flexion. This condition did further improve index finger extension. The total index finger extension angle, defined as the sum of the angles of the MCP, PIP and DIP, increased by 44.3 and 65.0 degrees in these two subjects relative to the unassisted condition.

## Discussion

Our analysis shows that when using SpringWear, subjects had significant gains in supination or pronation maximum, elbow extension maximum and shoulder flexion maximum during range of motion tasks. Subjects also integrated this new ability into functional task performance, where the maximum joint angles at these assisted DOF also increased. ROM increased in elbow FE during Task 2, but there were no significant changes in ROM in forearm PS or shoulder FE in any of the other Tasks. The initial starting positions of these two joints shifted with assistance, which prevented an increase in ROM even though the maximum joint angle increased in all joints. This change in initial position is undesirable, indicating that in the shoulder for example, an initial neutral position could not be achieved with assistance. It is possible that subjects had weakness in shoulder extension that prevented them from returning the arm to the neutral position against the springs. Alternatively, the assistance provided near neutral position was too large and a profile that drops more rapidly to 0 torque as the arm is dropped may be preferable.

For subjects receiving assistance in supination, returning to the unassisted maximum pronated position was not possible. This might have been due to the assistance profile that did not return to zero torque in full pronation as desired, because the springs needed to wrap around the forearm splint (note the discontinuity in the assistance profile in Fig. 3). Better performance may be possible with elimination of this discontinuity in the profile and a finer ability to adjust the levels of assistance. Currently, only discrete changes in the profile amplitude are possible, and the shape of the profile cannot be changed. We were surprised at how weak subjects were in this DOF overall, as the maximum torque applied across all subjects was 0.53 Nm, and yet subjects could not overcome this small torque to return to the initial joint angles achieved without assistance.

Four patients completed all functional tasks with SpringWear assistance, and also completed these tasks without assistance. Eight of nine patients who didn’t complete functional tasks without assistance also were not able to complete these tasks with assistance. Across all subjects, there is the significant increase in forward reach workspace (as shown in Table 4) which might be associated with less reliance on trunk compensation. The results from ROM tasks showed some improvement in finger extension ability with proximal assistance, particularly in the PIP joint. This is consistent with other studies that reported coupling between shoulder flexion and finger flexors [12, 13]. The shoulder flexion assistance may have decreased the effect of this coupling, allowing more finger extension. But these gains in extension may have been too small to make a difference in the functional tasks. Also from observations, four patients were able to open the hand large enough to grasp the object, but their grip strength was not adequate enough during the lift attempt. In the future, we would like to use SpringWear together with HandSOME to examine their combined effect on task performance.

Actively powered devices provide variable assistance that can be adapted to patient performance, can train a large variety of movement patterns and tasks and provide variable feedback [24–28]. In healthy subjects, these factors can have positive effects on motor learning and generalization to new tasks [28, 29]. However we are not aware of any clinical trials that support the hypothesis that these specific factors when integrated into training protocols for stroke patients, result in clinically important advantages over protocols that don’t include these factors. Another principle of motor learning is task specificity [5, 16]; learning a desired task is best accomplished by practicing that exact task under the same conditions that it will be spontaneously performed. The SpringWear is based on task specificity, expanding the range of user-driven movements and tasks that can be achieved, in the context of real-life tasks performed in the home, while also increasing the dose, realism and relevance of the task practice to the patient. Most powered robots require practicing components of functional tasks (ie: reaching or grasping) in isolation, in a seated position in the clinic, while viewing feedback on a computer monitor. In contrast, use of SpringWear in the home will allow patients to practice actual ADL of interest: bimanual tasks, reaching tasks while standing, grasp and manipulation of real objects in the home. Task variability can be incorporated simply by instructing the patient to use the arm in a variety of different tasks. This approach may carryover to increased spontaneous use of the limb more effectively than clinic-based training, addressing the evidence that adequate movement ability when observed in the laboratory does not necessarily correlate with regular incorporation of the limb into ADL [4]. While advances in powered devices will inevitably allow wearable versions to be used at home as well, SpringWear represents a first step to evaluate the potential of this approach with the benefits of a device with significantly reduced cost and complexity.

In stroke patients, many joints are imbalanced in flexion vs extension impairment [11], where one action is more impaired that the other. Elbow extension is much more impaired than flexion and SpringWear applies extension torque to balance the joint and increase ROM. The shape of the assistance profile also helps to increase ROM. An applied extension torque will clearly increase the maximum extension angle achievable. The applied extension torque decreases as the elbow is flexed, so that range in the flexion direction is maintained. This decrease in applied extension torque is important, as patients are often also weak in flexion and may not be able to overcome large resistance. In the case of shoulder flexion, a torque assistance profile that has the same shape as the gravity loading on the arm decreases the torque needed to flex the joint, increasing flexion range. As long as the torque assistance does not exceed the gravity loading on the arm, relaxing muscles allows the arm to fall back to neutral. An additional factor is the abnormal flexor synergy between the elbow and shoulder, whereby lifting the shoulder results in involuntary activation of elbow flexors. Studies have shown that increased elbow ROM can be achieved just with shoulder gravity support by weakening the effect of this abnormal flexor synergy [8, 10].

Recent studies show that the effectiveness of unassisted task-practice may plateau as dosage is increased. One study found no dosage effect in individuals with chronic stroke who received 3200, 6400, 9600 or 10,808 repetitions of upper extremity tasks [30]. A study of 361 individuals with subacute stroke found no differences between groups who received 28.3 h of intensive task oriented training, 26.7 h of occupational therapy, or 11.2 h of occupational therapy [31]. SpringWear allows practice of tasks with normal movement patterns, less reliance on compensation, less effort and reduced fatigue. Furthermore, because SpringWear is inexpensive, wearable and not tethered, it can be used at home during actual ADL. This paradigm may have advantages compared with traditional approaches that use practice of components of simulated ADL in the clinic.

Several spring based devices are now commercially available for gravity compensation for the shoulder. These include the ArmeoSpring (Hocoma AG, Switzerland), ArmeoBoom (Hocoma AG, Switzerland), and SaeboMAS (Saebo, Inc. Charlotte, NC). The ArmeoSpring has been tested most extensively, with clinical trials showing that significant gains are possible in chronic stroke patients [14, 32]. A controlled study showed advantages of using T-WREX (precursor to ArmeoSpring) compared to an active control group, particularly in followup testing [14]. SpringWear also provides gravity compensation, so should theoretically provide the same benefits as these other devices. However, SpringWear is different from these other devices in that it’s wearable, untethered, and targeted for use in actual ADL instead of with video game interfaces.

SpringWear does not accommodate scapular movement during the Scapular Humeral Rhythm (SHR). We based this design decision on the added complexity of a device that accommodates scapular movement and the expectation that the majority of shoulder flexion movements would be less than 90 degrees. Scapular movement increases as the humerus is elevated beyond 90 degrees but is smaller at lower flexion angles. However, consideration of the SHR will be needed for a device that accommodates shoulder flexion through its full normal range of motion. Loading the shoulder improperly can also disrupt the SHR and lead to negative effects such as shoulder subluxation. However, the use of an exoskeleton guarantees that the forces applied are perpendicular to the long axis of the humerus and are the minimum forces needed for gravity compensation at the shoulder and similar to the forces applied when a therapist assists movement of the joint. The spring force components parallel to the long axis of the humerus are not applied to the shoulder complex and are instead applied to the exoskeleton. Nevertheless, the effects on SHR should be examined in future studies and may be a potential reason why subjects were unable to return to neutral when wearing the device.

Studies have shown that training with passive gravity compensation devices are as effective as one-on-one conventional training with a therapist [33, 34]. We are not aware of any head-to-head comparisons of passive gravity compensation devices compared with powered robotic exoskeletons. While powered robotic exoskeletons have several theoretical advantages over passive devices, it remains to be seen if these theoretical advantages will translate into clinically important differences. Measuring the benefits of powered devices over passive devices is important, since passive devices such as SpringWear have advantages in terms of decreased cost and complexity.

### Limitations

A single camera and manual processing of images were used to measure the maximum index finger extension angles. Two dimensional video analysis has been reported to be comparable to the “gold standard” 3D motion capture for planar movements, and is a viable option for kinematic assessment [35–37]. We were careful to position the subject so that index finger movements were only in a plane parallel to the camera’s image plane. Note that we only analyzed data for the index finger and did not attempt to measure movements in the other fingers or the thumb. However, this approach could lead to errors if there is out of plane motion and in the future we plan to use 3D video motion capture of finger movement to measure the grasp aperture during movement. Comparison to the grasp aperture used by healthy controls in the functional tasks would support the theory that inability to open the hand limited the subject’s ability to complete the functional tasks.

The manual procedure used to customize the spring stiffness to the impairment level should have enabled subjects to voluntarily hold an arm position of shoulder flexion to 90 deg., elbow fully extended and forearm supinated. As patients recover over the course of many weeks, we envision this manual procedure can be repeated regularly and lower stiffness values used to force patients to contribute more to the movements. Spasticity can result in changes in patient performance over the course of minutes, so manual adjustment of stiffness to compensate is not practical. However, the effects of increased spasticity will be apparent to the patient in decreased movement ability, and this performance feedback might be useful in learning to overcome spasticity. In contrast powered robotic devices with adaptive assistance algorithms would overpower the spasticity and complete the movements for the patient, which might be counterproductive and only worsen the spasticity.

More work is needed to find the optimal assistance profile for each subject. We tested the device with assistance levels that we thought would produce the largest gains in ROM. We added gravity compensation assistance at the shoulder until the subject could voluntarily hold the shoulder at 90 deg. of flexion. Therefore the effort level at the shoulder was still very high in our testing. When the assistance was increased to 100% gravity compensation in 2 subjects, maximum index finger extension increased further. However, under these conditions, the subjects ROM was severely limited and subjects were unable to extend the shoulder back to neutral. A level of assistance between these two extremes might have yielded better results. The bulky appearance is a negative and can in some cases interfere with activities. Also, a caregiver is required to assist with donning the device. More work is needed to streamline the design and improve usability.

Future studies should expand the range of functional tasks that can be tested. The tasks we chose were based on components of the Action Research Arm Test (ARAT) [38]. The ARAT involves picking up objects and replacing them on the table. Our goal was primarily to determine if the reaching component of reach and grasp tasks was improved by wearing the device. Since changes in hand function were expected to be minor with the device, we did not include the larger array of objects used in the ARAT to test specific hand functions. Instead, we selected 2 tasks that were most likely to improve when wearing SpringWear. We chose the water bottle task because the gravity compensation assistance may reduce the negative effects of the weight of a fairly heavy object, such as a full water bottle. We chose the dowel task because it required almost full range of motion at the supination/pronation DOF to complete, and so the assistance at this DOF provided by SpringWear may have a large impact. A more systematic and complete test of functional performance would be achieved with a standard clinical test such as the ARAT or the Sollerman hand function test [39].

## Conclusions

The SpringWear increased shoulder flexion, elbow extension and forearm PS maximum values both during ROM testing and functional tasks. Elbow FE ROM was also significantly increased. More work is required to understand why patients were unable to return to neutral positions in shoulder FE and forearm PS joints when assisted. Nevertheless, ROM was not decreased in any instance and the shift of useable ROM to more functional values may prove useful. No consistent improvement in the ability to complete functional tasks was noted, and this was likely due to only small improvements in hand movement with proximal assistance. Use of HandSOME with SpringWear may yield better results during functional task practice and help to overcome the problem of abnormal synergies linking distal activation of flexor muscles when flexing the arm against gravity. These low-cost devices can potentially reduce effort and improve performance during ADL, increasing adherence to home training programs for rehabilitation.

## Abbreviations

ADL: Activities of daily living
deg.: Degree
DIP: Distal interphalangeal joint
DOF: Degree of freedom
FE: Flexion/extension
HandSOME: A wearable passively actuated hand exoskeleton
MCP: Metacarpophalangeal joint
PIP: Proximal interphalangeal joint
PS: Pronation/supination
ROM: Range of motion
SD: Standard deviation
SpringWear: Spring Operated Wearable Enhancer for Arm Rehabilitation
